# The quest for knowledge transfer efficacy: blended teaching, online and in-class, with consideration of learning typologies for non-traditional and traditional students

**DOI:** 10.3389/fpsyg.2014.00324

**Published:** 2014-04-17

**Authors:** Judy R. Van Doorn, John D. Van Doorn

**Affiliations:** ^1^Psychology Department, Troy UniversityPhenix City, AL, USA; ^2^International Relations Department, Troy UniversityColumbus, GA, USA

**Keywords:** blended/hybrid courses, online courses, face-to-face (f2f) courses, learning needs typology, non-traditional students, traditional students, knowledge transfer, student learning outcomes SLOs

## Abstract

The pedagogical paradigm shift in higher education to 24-h learning environments composed of teaching delivery methods of online courses, blended/hybrid formats, and face-to-face (f2f) classes is increasing access to global, lifelong learning. Online degrees have been offered at 62.4% of 2800 colleges and universities. Students can now design flexible, life-balanced course schedules. Higher knowledge transfer rates may exist with blended course formats with online quizzes and valuable class time set for Socratic, quality discussions and creative team presentations. Research indicates that younger, traditional students exhibit heightened performance goal orientations and prefer entertaining professors who are funny, whereas non-traditional students exhibit mastery profiles and prefer courses taught by flexible, yet organized, professors. A 5-year study found that amongst 51,000 students taking both f2f and online courses, higher online failure rates occurred. Competing life roles for non-traditional students and reading and writing needs for at-risk students suggest that performance may be better if programs are started in f2f courses. Models on effective knowledge transfer consider the planning process, delivery methods, and workplace application, but a gap exists for identifying the diversity of learner needs. Higher education enrollments are being compromised with lower online retention rates. Therefore, the main purpose of this review is to delineate disparate learning styles and present a typology for the learning needs of traditional and non-traditional students. Secondly, psychology as a science may need more rigorous curriculum markers like mapping APA guidelines to knowledge objectives, critical assignments, and student learning outcomes (SLOs) (e.g., online rubric assessments for scoring APA style critical thinking essays on selected *New York Times* books). Efficacious knowledge transfer to diverse, 21st century students should be the Academy's focus.

## Introduction

The pedagogical paradigm shift in higher education to 24-h learning environments, encompassing several delivery formats including online courses, blended/hybrid designed courses, and the traditional face-to-face (f2f) lecture classes have increased student access and engagement into global, lifelong learning. The Babson Survey Research Group, in a 10-year study, found that fully online degrees have been offered at 62.4% of the 2800 colleges and universities surveyed and the results indicated an increase of over 6.7 million students taking at least one online course during Fall of 2011 (Allen and Seaman, 2013; Estes, 2013). Teaching pedagogy is dramatically changing within today's challenging educational environments, composed of diverse traditional, and non-traditional student populations. Many educators are experiencing the benefits of greater flexibility in course design and in delivery methods by building courses with technology infused tools. The traditional pedagogical lecture method of f2f delivery, with all content delivered in the classroom, transforms into a web-facilitated course when technology tools like course management systems (CMS) and teacher designed web pages are used to enhance teaching delivery. Asynchronous online courses are now considered the most common delivery format, although teacher and student feedback is delayed. Another teaching style includes the blended/hybrid course format consisting of combinations of f2f teaching, synchronized (real time feedback), and asynchronous online delivery (Dunn et al., 2011b). Also, there is the “flipped” type model where students watch taped lectures outside of classroom time. Lifelong learning is being promoted through continuing education programs to attract new students and improve retention with a goal of widening participation across diverse populations (Roberts, 2011; Caffarella and Daffron, 2013). Free massive open online courses (MOOCs) are being offered at prestigious universities, with completion certificates offered at 2.6% of universities, and they are part of strategic plans in another 9.4% (Allen and Seaman, 2013; Christensen and Horn, 2013; Phillips, 2013). However, a University of Pennsylvania research study found a completion rate of only 4% with MOOCs (Lewin, 2013).

Present research is painting a mixed portrait of student performance and their university experience that varies across pedagogical delivery methods. Prior research by Daffron and North (2011) has indicated the need of the Academy to focus on the efficacy of successful knowledge transfer. In their Transfer of Learning Model, seven variables are considered including (1) the planning process, (2) learner characteristics and motivation (3) design and delivery methods, (4) learning context, (5) immediate application, (6) workplace environment, and (7) eliminating barriers (Daffron and North, 2011; Caffarella and Daffron, 2013). While this model addresses transfer process influencers and planning processes, a gap still exists for identifying the diversity of learner needs. Higher education administrators are finding that enrollments are being compromised with lower online retention rates and subpar student performance. Educational administrators may overly focus on per student cost approaches to education vs. focusing on performance outcomes and learner needs matched to delivery methods and educator abilities. Therefore, the main purpose of this research review is to delineate disparate learning styles and present a typology for the different learning needs of traditional and non-traditional students in higher education.

Foremost, typical university student populations have dramatically changed and are now composed of differences in demographic characteristics, socio economic status (SES), part-time and/or full-time student workers, and military cohorts. The traditional student (ages 18–21 years) generally works part-time, attends day classes, and participates in the university social experience; whereas an emerging “new student” cohort is the non-traditional, adult student (ages 22–55+ years) who works more full-time, juggles family responsibilities, and attends more night courses (Munro, 2011). Additionally, we propose that psychology as a science and other social science disciplines may need to update, to improve scholarship in teaching and performance outcomes, and to consider developing more rigorous assessments within faculty-approved curricula (American Psychological Association, 2007, 2013; Buskist et al., 2008).

Clearly, it is beneficial for students to have the flexibility of at least some online course delivery for their degree program. However, research on the learning efficacy of different delivery methods is starting to reveal some associated complexities, including lower retention rates, and maybe lower levels of content mastery. Alarming university dropout rates, coupled with the need for student retention, has led to research evidence suggesting that different student profiles do exist, and maybe these student cohorts have culturally different ways of learning. Columbia University's Community College Research Center conducted a 5-year study and found that among 51,000 students taking both f2f and online courses, higher failure rates occurred with the students who took online courses. Also, lab-based social science courses, including psychology, were rated as more difficult for students to take online. Non-traditional students, with competing life roles and at times considered at-risk students who have higher needs for reading and writing instruction, may perform better in high fidelity, traditional f2f courses (New York Times - Editorial, 2013; Xu and Jaggars, 2013). Students who need basic math and English skills may need more guidance from their teachers, using a scaffolding learning approach (Vygotsky, 1978). Furthermore, Xu and Jaggars (2013) found significant discontinuities in online learning between different demographic groups and suggest four solutions: (1) delay online courses for screened, at-risk individuals, (2) start learning support approaches like scaffolding, (3) use online warning systems for low performance (e.g., Starfish), and (4) pedagogical focus on building quality online courses. Although there is an exciting growth of global MOOCs and other fully online course programs, there seems to be research gaps on measuring student behavior, performance, and motivation while taking online courses, along with measuring their persistence in completing courses, and content mastery within the social science disciplines.

Educational researchers need to refocus on the different pedagogical models being experienced and driven by a surge of technology-based online classes. Not only do students have greater flexibility in designing program schedules with f2f and/or online learning environments, but also differences in traditional and non-traditional learning styles and needs challenge teachers to adapt their teaching methodology. Many non-traditional, working students describe “ideal” professors as flexible and organized; whereas, traditional students describe them as funny and enthusiastic (Rosenthal et al., 2000; Strage, 2008). Learning styles differ such that the profiles of mature, non-traditional students who transfer from community colleges to universities exhibit more performance mastery of content, while younger traditional students show more performance-goal orientations (Dweck, 1999; Hoyert and O'Dell, 2009). Non-traditional students tend to value more educational, lifelong learning goals, and are mindful of the potential socio-economic benefits associated with higher learning; whereas, younger, traditional students are more reflection-oriented about their educational experience and focus on the performance of making high grades (Jinkens, 2009). In order to address the research disparity and clarify learner differences, this literature review and proposed typology is guided by category questions that delineate the disparate learning styles of traditional from non-traditional students in hopes for higher academic performance and residual, retention goals. Our literature research review is guided by seven categories that address different traditional and non-traditional student needs and are as follows:

1. What are the different learning needs?
2. What institutional support is needed?
3. What are the computer technology needs?
4. What are the educational culture and social needs?
5. What faculty-matched abilities are needed?
6. What learning styles (auditory, visual, and/or kinesthetic) are needed?
7. What is needed for different course subjects?

Through these directed research questions and supporting research evidence, the main purpose of the review is to delineate disparate learning styles and present a distinct student learning needs typology, created as a working reference, for traditional and non-traditional student cohorts across course delivery formats. Secondly, we propose that higher knowledge transfer efficacy may be achieved in support of psychology as a science through building more rigorous curriculum markers like mapping APA guidelines to knowledge objectives, critical assignments, and student learning outcomes (SLOs). Thirdly, we review learner needs across the course delivery methods of f2f, blended/hybrid, and fully online formats. The hope is that administrators and educators may be able to increase student performance and retention rates by matching teaching delivery methods to the disparate learning needs of the 21st century university students.

### Non-traditional and traditional student learning styles

Many researchers in teaching pedagogy have focused their studies on the academic needs of the traditionally perceived student (ages 18–21 years) who usually attends daytime courses in college classrooms, does extensive library study, and stays to participate in social activities on university campuses (Munro, 2011). Another growing academic student cohort is composed of the non-traditional, adult student-workers (22–55+ years of age) who usually attend more night time classes at satellite campuses, and balance more competing life roles including family, elderly care, and multiple part-time to full-time jobs along with their academic schedules. International and respective American literature define non-traditional students at the undergraduate and graduate levels as (1) individuals who enter school several years later after high school at 23 or 25 years old and may need preparatory math or English reentry courses, (2) minority students from different ethnic backgrounds with lower socio-economic status and/or (3) individuals who may have risk factors for dropping out of school including full-time employment, multiple family role responsibilities, and single parenthood (Kim and Bonk, 2006; Miller and Hudson, 2007; Gilardi, 2011; Xu and Jaggars, 2013). A Pew Research Center study found African Americans Internet use is 80% compared to the 87% use by whites indicating a 7% gap in technology access (Smith, 2014). Also, 73% of African Americans who use the Internet also use social networking platforms, like Twitter and Facebook. Although, the research revealed that 92% of African Americans do own cell phones of which 56% are smartphones, giving this cohort potentially equal access to courses through the use of their mobile platforms. Therefore, universities need to make sure computer labs are available with day and night hour access especially for non-traditional students.

Recent studies are addressing non-traditional student time management, stress and coping factors (Forbus et al., 2011), and their attitudes and performance with online distance learning (Beaghan, 2013). Research from Australia, the United Kingdom, and the United States on how the non-traditional student-worker, or emerging “new student” in UK literature, faces academic challenges which include performing quality academic work and fitting into the university environment (Munro, 2011). Research evidence suggests that only a minority of Australian working-class individuals, who had been exposed to fewer opportunities and more educational exclusions, were able to gain a university education (Pearce et al., 2008). This research found three themes existed for the educational success of non-traditional students including (1) self-discovery joy of the world, (2) a mission or quest to make the world a better place, and (3) a chance to make better lives for themselves and their children. The qualitative narrative research of Pearce et al. (2008) found that successful non-traditional students were persistent and would not give up on fitting in and attaining a higher education. Furthermore, these researchers advocate “second chance” programs for the “unfinished business of schooling.”

Gilardi (2011) researched students of Political Science across the northern Italian public university system and found that non-traditional students focus more on the learning experience and do have more difficulties as compared to traditional students in navigating the university system the first year. Also, Italian and USA literature reviews supported some of these differences between traditional and non-traditional students such that traditional students have a greater need for learning context and meaningfulness associated with the university experience. On the other hand, evidence found that non-traditional students need traditional in classroom experiences and value their education as part of professional development. This career development focus helps them connect their own professional life experiences to learned theory, and decipher many implications on an applied level (Gilardi, 2011). Van Doorn and Chesterman (2012) suggest that non-traditional students “raise the bar academically” through their wisdom gained from life experiences and transfer this vicarious benefit to traditional students through collaborative group work in class. Also, the traditional students bring their youthful enthusiasm and knowledge of current social and cultural trends to classroom sharing, in turn, benefiting non-traditional students. However, it is a noteworthy fact that only a minority of students has access to living on college campuses (Christensen and Horn, 2013).

United States community colleges serve half of all undergraduate students with over 6.5 million enrolled in 2005. Community College serve 68% white students, 27% black, non-Hispanic, 1% Hispanic, 1% American Indian, 1% Asian-Pacific Islander, and others (American Association of Community Colleges, 2014). International student enrollments in American colleges for fall 2009 increased by 3% and were mostly represented by students from China, India, the Middle East, and Africa (Fischer, 2010). Hermida (2010) expands the non-traditional student definition by including recent immigrants, internationals, and first-generation students and challenges the North American pedagogy as less integrative of the culture, diversity, and values of non-traditional students. Furthermore, this research evidence suggests offering a pedagogical strategy of “inclusive teaching” with diverse knowledge modes and the use of the expressive story-telling style. Therefore, the entire student classroom gains in heightened diversity perspectives from this story-telling technique that demonstrates peer cultural differences (Reevy, 2012). In class storytelling fits better with in class delivery, but also could be adapted through video taping student stories to upload in the online class. Additionally, traditional students tend to value organizational support when integrating into a university and represent 60% of the student population; whereas, the other 40% composed of non-traditional students value the overall academic environment for their university commitment (Wardley et al., 2013).

There are several key differences between the learning styles and needs typology of traditional vs. non-traditional students. Firstly, traditional students are more ready to enter university with more confidence usually due to working less than 40 h in part-time employment and by having considerably fewer family responsibilities to interfere with academic studies. On the other hand, many years may have passed since the non-traditional student was last in a formal learning environment and may need to build personal confidence and motivation to not only return, but also to persist within the educational culture. Also, non-traditional students may have very full daily schedules and, thereby, experience time management constraints and fatigue issues. Late night course offerings, whether f2f or online, may be the best options for non-traditional students. Evening courses may be best delivered through a mix of teaching methods of lecture, video, group work, and role-play in order to keep a lively, upbeat discussion that fully engages students who have just arrived from a full day of work. In addition, institutional integration and social connections may be met by club memberships (e.g., Psi Lambda psychology club, Psi Chi International Honor Society, Pi Sigma Alpha). Outside-the-classroom learning opportunities may involve service-learning opportunities like community volunteerism, charity fundraising, applied work internships, and job networking. Most traditional students prefer the university social experience of sororities and fraternities; whereas non-traditional students who are achievement-oriented have less daytime available for social events.

This brings us to a discussion of how to best address the very different needs and learning styles of traditional vs. non-traditional undergraduate and graduate students. We suggest that research is revealing distinct learning style differences in these student cohorts. Mapping pedagogical delivery formats and university support services to these distinct need differences may enhance overall student educational experiences for improved retention and learning mastery. Our typology, as shown in Table 1, for traditional and non-traditional student learning needs are categorized into seven sections including for (A) learning needs (B) institutional support, (C) computer technology, (D) educational culture, and social needs, (E) faculty-matched abilities, (F) learning style considerations, and (G) course subject needs. The student needs are compared between traditional and non-traditional needs based on research findings and matched by number. For example, the learning need for traditional students includes (1) an entertainment-style of teaching with maximum in-class time and personal contact with enthusiastic instructors; whereas, non-traditional students prefer (1) organized classroom structures with some online instruction (Hoyert and O'Dell, 2009). See Table 1 for the complete typology of parallel comparisons between traditional and non-traditional student learning styles and needs.

**Table 1:** **Typology of traditional and non-traditional student learning styles and needs**.

**Traditional undergraduate students** (*Graduate student needs)	**Non-traditional undergraduate students** (*Graduate student needs)
**LEARNING NEEDS**	
Prefer entertainment style of teaching, maximum in-class time, and personal contact with enthusiastic teachers (Rosenthal et al., 2000; Hoyert and O'Dell, 2009)	Prefer flexible, yet organized teachers and organized classroom structures Rosenthal et al., 2000; Hoyert and O'Dell, 2009
Performance goal-oriented Hoyert and O'Dell, 2009	Subject mastery-oriented Hoyert and O'Dell, 2009
Changing needs for program course flexibility (e.g., use of video media like Youtube.com for assignments, interactive blogs, and social networks)	Availability and greater use of support services for learning Gilardi, 2011
Share cultural and current trends in class Van Doorn and Chesterman, 2012	Share work experience wisdom and diversity differences in class Van Doorn and Chesterman, 2012
Participate in Inclusive Teaching like story telling techniques to facilitate learning with non-traditional peers Hermida, 2010; Reevy, 2012	Participate in Inclusive Teaching like cultural story telling techniques to learn difficult concepts and connect with traditional students Hermida, 2010; Reevy, 2012
High participation in activities in the classroom to reduce attrition and improve attendance Gilardi, 2011 (e.g., sorority and fraternity membership, campus service clubs)	Lively discussions and group work in class due to full-time work stress and fatigue Forbus et al., 2011
“*Digital natives*” with need for use of all technology and social media platforms American Psychological Association, 2013	^*^Novices or “luddites” in need of technology training and use of course delivery formats Dunn et al., 2011a,b
^*^High need for program course flexibility to fit part-time work schedules: F2f, online, web facilitated, and blended/hybrid courses	^*^High need for program course flexibility to fit full-time work schedules: F2f, online, web facilities, and blended/hybrid courses
^*^In-person access to large on-campus and online research library, archives, databases, research laboratories, and search engines (e.g., Surveymonkey.com, Qualtrics.com)	^*^Access to online research library databases Some access to research search engines (e.g., Surveymonkey.com, Qualtrics.com)
Prefer online courses and some f2f courses with opportunities for supplemental on-line discussions (web facilitated and blended/hybrid formats)	Prefer f2f courses with some opportunities for supplemental on-line discussion (web facilitated and blended/hybrid formats)
^*^Learning models that incorporate more diversity American Psychological Association, 2013	^*^Learning models that incorporate more diversity American Psychological Association, 2013
^*^Maximum working time with supervisor during open laboratory hours	^*^Limited time with research supervisor during planned office hours
^*^National and International Conference research presentation needs	^*^Regional conferences for convenience—research presentation opportunities
^*^On campus research assistantships, work internships, and/or volunteerism for applied learning	^*^Off campus part-time internships, research assistantships, and/or volunteerism for applied learning
**INSTITUTIONAL SUPPORT NEEDS**	
University learning experience need with meaningfulness, resulting in higher retention Gilardi, 2011	University integration and relationship building with faculty, may result in higher retention (Gilardi, 2011)
Organized advising and program evaluation at key times including first semester planning, mid-program, and senior graduation year Kirp, 2014	More institutional, faculty, and advisor support for learning and social integration throughout degree program (Most et al., 2013; Beck and Milligan, 2014)
University support for self-discovery; campus activities and events for quick social integration Pearce et al., 2008	“Second chance”—the unfinished business of school programs to improve life chances and confidence building Pearce et al., 2008
Graduate school planning and career placement needs	Career placement and professional transition needs Some graduate school planning needs
**COMPUTER TECHNOLOGY NEEDS**	
More daytime computer lab and social media access while on campus Smith, 2014	More nighttime computer lab access—preferably 24-h computer labs Smith, 2014
BYOD, bring your own device; connection outlets for use of personal technology on campus Smith, 2014	Technology access to iPads, eReaders, and mobile platforms and use of Smartphones Smith, 2014
**EDUCATIONAL CULTURE AND SOCIAL NEEDS**	
First year integration into educational culture through student organizations (Reay et al., 2010; Munro, 2011; Kirp, 2014)	“Fitting in” educational culture and confidence building (Reay et al., 2010; Munro, 2011; Kirp, 2014)
Student identification, but appreciation building for the non-traditional student cohort Gilardi, 2011	Need for social opportunities, yet flexibility to lower attrition rates Gilardi, 2011 (e.g., service learning activities, club opportunities)
“Fitting in” and learning to become a critical thinker Munro, 2011	Reclassification as the emerging “new student” Munro, 2011
^*^Study Abroad—long term stay; global learning opportunities American Psychological Association, 2013	^*^Study Abroad—short term stay; global learning opportunities American Psychological Association, 2013
**FACULTY MATCHED ABILITIES**	
Social Media trained instructors (Smith, 2014); Online course training Quality Matters Program, 2011	Faculty with professional work experience; Online course training Quality Matters Program, 2011
Entertaining lecture style Hoyert and O'Dell, 2009	Faculty adept at in class pragmatic learning techniques Nemanich et al., 2009
Faculty knowledge of diverse student populations American Psychological Association, 2013	Faculty knowledge of diverse student populations American Psychological Association, 2013
Mentoring and advising duties	Mentoring and advising duties
Faculty knowledge of cognitive memory styles Sternberg and Grigorenko, 1997 for *training* students on study skills	Faculty knowledge of cognitive memory styles Sternberg and Grigorenko, 1997 for *relearning* of study techniques
Visual Imagery and word associations (Shepard, 1967; Paivio, 1969; Reed, 2013)	Visual Imagery and word associations (Shepard, 1967; Paivio, 1969; Reed, 2013)
Mnemonics Lorayne and Lucas, 1974	Mnemonics Lorayne and Lucas, 1974
*Training* students on Goal-setting theory and task motivation; rebuilding student self-efficacy Locke and Latham, 2002; Bandura and Locke, 2003	*Retraining* students on Goal-setting theory and task motivation; rebuilding student self-efficacy Locke and Latham, 2002; Bandura and Locke, 2003
	Use of scaffolding learning techniques Vygotsky, 1978
**LEARNING STYLES: (VISUAL, AUDITORY, AND TACTILE/KINESTHETIC;**	
Visual Learning: use of visual videos, lectures, teamwork	Mixed methods used in class: videos, written assignments, lectures, group work
Auditory learning: taped online lectures for “flipped” and online	In class experiential and kinesthetic Style demonstrations; blended/hybrid facilitated
Kinesthetic learning: visits to museums, environmental learning, and space centers	Auditory learning through online voice-over lectures Weiermann, 2012
**COURSE SUBJECT NEEDS: (PRELIMINARY LIST)**	
Math—group study sessions for math problem practice	Math—offer in class courses with available math tutors
English—experiential learning (attend plays)	English—access to writing labs
Psychology—access to ^*^research labs	Psychology—relate material to practical work world; in class or online labs
International relations—model United Nations participation.	International relations—^*^simulations with experiential learning and scenario-based case studies
Business—entrepreneurial projects; organizational field studies	Business—pragmatic case studies

### Knowledge transfer strategies and learning styles

Research indicates that individuals learn differently through their physical senses of visual and auditory modalities and the tactile touch sense of kinesthetic learning. Educators who match their teaching methods to visual, auditory, and kinesthetic learners will enhance their students' knowledge transfer processes (DeBoth and Dominowski, 1978; Korenman and Peynircioglu, 2007; Weiermann, 2012). Research on comparing the efficacy of online training vs. f2f reveals that both increase social capital, but that the computer supported collaborative students had gained more group observation skills (Mebane et al., 2008). Research evidence indicates that the dynamic between instructor expertise and social richness of an in class environment enhances student enjoyment (Nemanich et al., 2009). In class course enjoyment was positively related to learning performance, but not online. In contrast, student's ability was positively related to online performance.

Educators may need to systematically teach cognitive methods to train learning skills with students, especially for non-traditional students in need of relearning study techniques. Robust cognitive learning strategies including mnemonics (Lorayne and Lucas, 1974), visual imagery, and word associations, (Shepard, 1967; Atkinson and Shiffrin, 1968; Paivio, 1969, 2008; Reed, 2013) and self-regulation learning strategies of forethought, performance, and self-reflection help with learning. Computer training can build technology self-efficacy to aid in online learning (Murray, 2000; Sitzmann, 2011; Wang et al., 2013). Support services including writing labs and math tutor services are value-added learning centers that may assist at risk students and non-traditional students in need of writing practice. These learning strategies have been found to enhance student performance and cognitive learning in the academic environment.

**Figure 1:**
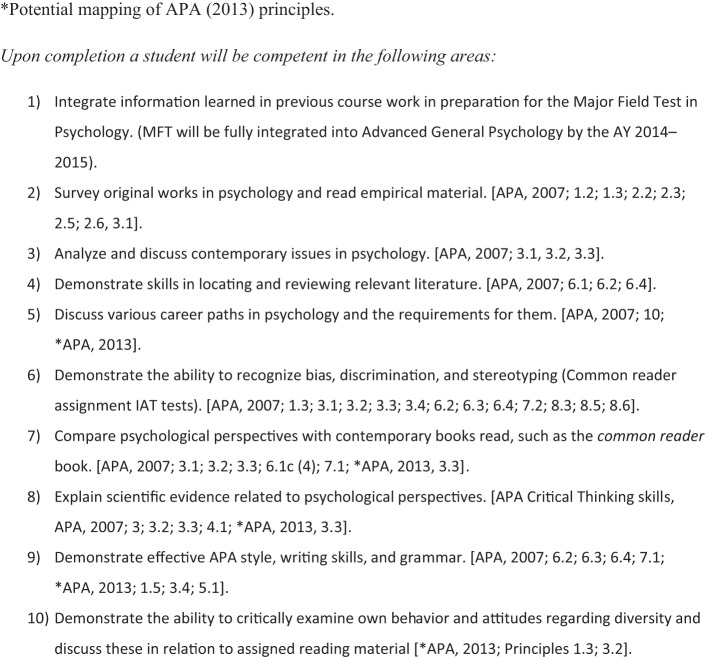
**Example of student learning objectives for senior seminar course with mapping to APA guidelines (American Psychological Association, 2007, 2013; Undergraduate Psychology Curriculum Committee, 2013).** ^*^Adapted mapping of American Psychological Association (2013) principles. ^*^Potential mapping of American Psychological Association, (2013) principles.

**Figure 2:**
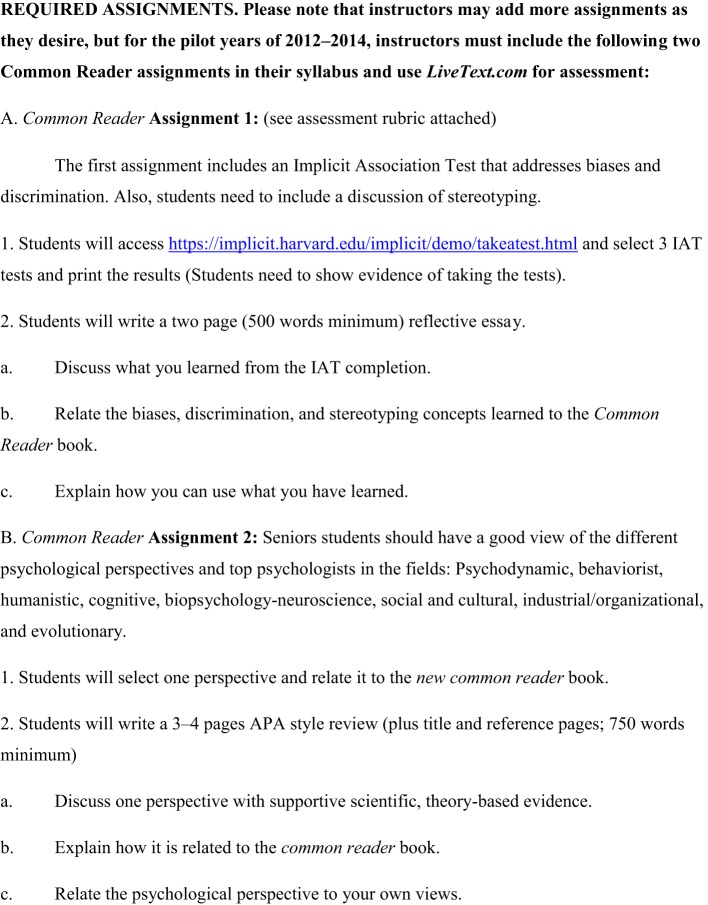
**Example of common reader assignment instructions (Undergraduate Psychology Curriculum Committee, 2013)**.

**Figure 3:**
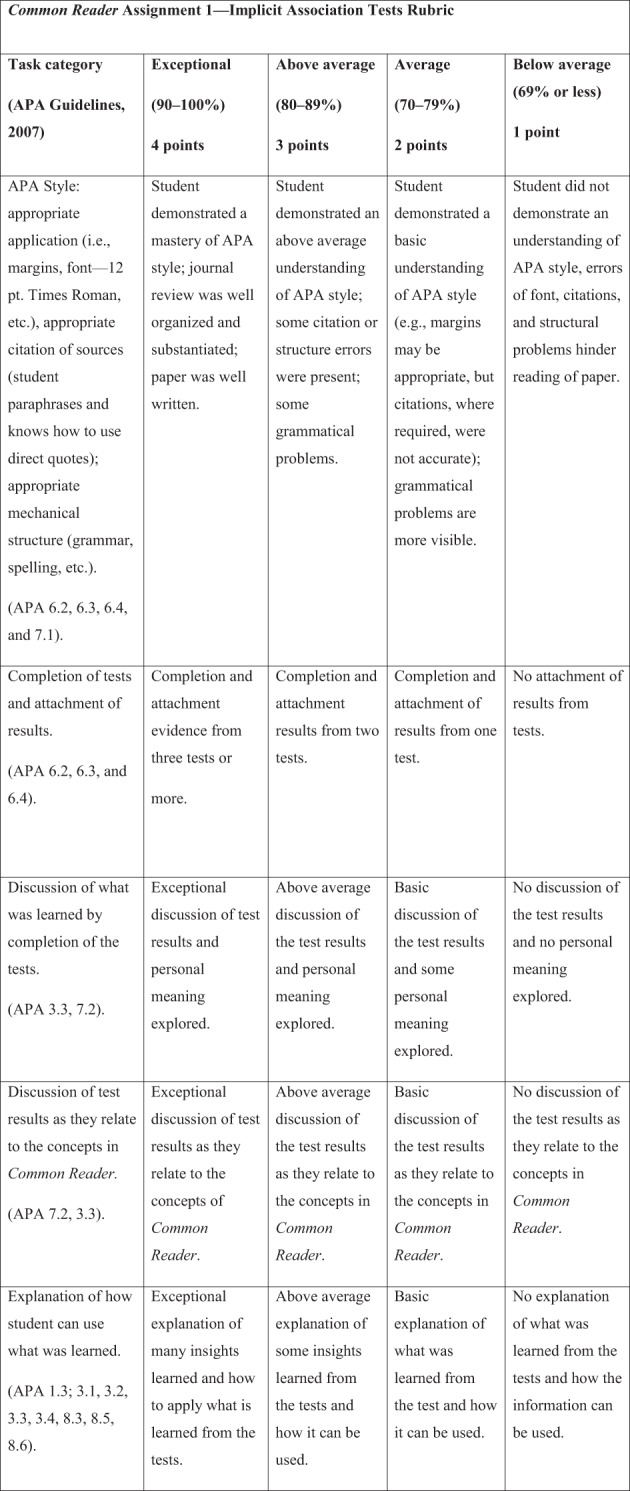
**Example of rubric for common reader assignment 1 on *Livetext.com* (Undergraduate Psychology Curriculum Committee, 2013)**.

Traditional students are considered “*digital natives*” who have grown up in the global information age using computers to listen to music and to communicate (American Psychological Association, 2013). Technology-savvy students increase the demand for faculty to enhance their science of learning delivery to students as recommended by Principle 2, number 8 of the APA *Principles for Quality Undergraduate Education in Psychology* (American Psychological Association, 2013). Faculty-led curriculum steering committees could design web-facilitated, common assignments as course supplements that are posted within online core course shells. These common assignments can be mapped to the disciplinary APA guidelines for the undergraduate psychology major (American Psychological Association, 2007, 2013; Undergraduate Psychology Curriculum Committee, 2013) and to student knowledge objectives, critical thinking skills, and course learning outcomes. See Figure 1 for an example mapping of an undergraduate psychology curriculum committee (UPCC) list of SLOs mapped to APA guidelines and principles (American Psychological Association, 2007, 2013; Undergraduate Psychology Curriculum Committee, 2013). For example, faculty may incorporate rubric-based, online programs like Livetext (www.livetext.com) and/or use CMSs like Blackboard for students to upload critical thinking essays written on selected *New York Times* best-seller books as common readers. With this type of assignment faculty can evaluate student mastery of APA style writing which may improve SLOs, while also providing assessment data evidence for university accreditation purposes. See Figures 2, 3 for an example of an online Livetext assignment with a grading rubric attached (Undergraduate Psychology Curriculum Committee, 2013). Suggestions for common readers and selected by the UPCC included *Blink: The Power of Think without Thinking* (Gladwell, 2004) and *The Noticer: Sometimes, all a person needs is a little perspective* (Andrews, 2009).

As university administrators feel the competitive pull from fully online educational institutions, strategies for providing best practices and methods in teaching are being reviewed and updated, especially with the revealing low retention and completion rates found for online learning with MOOCs (Lewin, 2013). Some online-only educational providers have been criticized for less rigorous programs by offering courses that are not accredited. The U.S. Department of Education lists all accredited programs (U.S. Department of Education, 2014). Students who attend unaccredited colleges may compromise education quality and their careers when they face employers who may deny their degrees, find credits are nontransferable to accredited institutions, and have less access to government loans (Cooper, 2014). Universities are confronted with what makes them unique, academically rigorous, accredited, and thus more valuable to students as they increasingly move to online courses. This dilemma is captured in Doonesbury comic strips (Trudeau, 2011) where students float in cyberspace between interchangeable online schools at http://www.gocomics.com/doonesbury/2011/07/17. On another note, an institution's educational face validity—portrayal of student investment in quality learning—needs to match the rigor of accredited degree programs experienced by students. Furthermore, educational institutions may need heightened consideration of the importance of the experiential value of being a campus student and the social support functions associated with university membership for adult learners (Lundberg et al., 2008). With the typology of traditional and non-traditional student learning needs compared and curriculum rigor considerations, the delivery modalities need to be clearly reviewed and discussed for the teaching nuances found in each format of f2f, blended/hybrid, and fully online course. A brief review will follow in the next sections identifying key differences between the delivery methods.

## Teaching styles

### Traditional face-to-face format

Classic teaching guidance for newly minted teachers emphasized the need to transform how students observe world environments—a social constructivism approach. This approach uses the processes of perception, analyses, and expression found in classroom debates where students critique and analyze the issues with their peers by engaging in discussions, laboratory sessions, collaborative learning, and field trips (Vygotsky, 1978; Wilkinson, 1984; Brufee, 1999). The art of teaching and classroom dynamics are rapidly changing with new technology-enhanced learning tools (Dunn et al., 2011a,b). Traditional f2f courses are defined as in class courses where content is taught in the classroom without any online technology, and course work is completed through writing assignments, exams, and homework (Allen and Seaman, 2013). Talented educators tend to elucidate core concepts, clearly articulate expectations, and transfer enthusiasm for the subject in a brief, efficient, and high fidelity manner. Within traditional f2f classrooms, a majority of students can have their questions answered instantly all at once vs. the delayed response times on asynchronous online courses.

The nuances of communication in facial expressions between students, peers, and instructors are part of the in class learning process for building relationships, career networking, and enhancing social and emotional intelligence (Goleman, 1995; Kristjαnsson, 2006). The classroom is a high fidelity learning environment where individual kinesthetic senses and perceptions are heightened. Instructors and students can actively conduct role-plays, student presentations, debates, and round-robin discussions (Van Doorn et al., 2012). Round-robin discussions involve taking a key idea and passing it along to another group to elaborate finer points. Here students invoke their personal style of voice and culture through communication, interpretation, and presentation skills. Additionally, teachers may find f2f learning provides unmatched learning opportunities enhanced with student participation in fun, exciting learning exercises like Jeopardy! Quiz Games and the use of Clickers (Personal Response systems) for instant feedback (e.g., polling and concept development). Jensen (2011) found students like the convenience of online video lectures, but prefer attending traditional lectures suggesting they have more focused attention in class. Classic psychology experiments may be remembered better if performed as demonstrations and as hands-on laboratory work. Research by Chen et al. (2009) found evidence that nuanced lessons conducted outside increased naturalist intelligence of students (Chen et al., 2009). Student/teacher and mentor relationships may be formed with direct access to professors for advisement and out-of-class (OCC) communication (Dunn et al., 2011a,b). Advisors can and do counsel students on course selection in order to design balanced schedules of in class and online courses that better fit busy life styles. And, f2f advising helps monitor students and their program evaluation progress for successful course completion to graduation. Researchers have found that thoroughly advised students have better learning attitudes and also have higher views of teachers as caring and competent. Also, they rate teachers higher on their evaluations (Clark et al., 2002; Dobransky and Frymier, 2004; Myers, 2004).

## Web facilitated and blended/hybrid course formats

In the Babson survey report, Allen and Seaman (2013) describe a web-facilitated course as a f2f course with about 1 to 29% of course content delivered online with use of classroom technology, while the definition of a blended/hybrid course delivery method includes about 30 to 79% of course content delivered online through CMS (i.e., Blackboard, eCollege, Desire2Learn, Canvas). Research indicates that blended/hybrid courses may be the best teaching format for a variety of student learning styles, because of its combination of f2f lectures and web-facilitated learning environments (Mansour and Mupinga, 2007). Higher student learning may result from blended course formats where most quizzes and exams are delivered online, therefore, allowing professors to save valuable class time for quality discussions, creative team presentations, and use of the Socratic method for concept clarification. Columbia University's Community College Research Center found evidence for the blended/hybrid delivery format as being equal to the performance of traditional lecture, f2f courses (New York Times - Editorial, 2013). Blended/hybrid courses demand organized synchronous (real time) delivery by professors along with asynchronous online delivery. Some university accreditation guidelines require courses be delivered with specific time ratios of synchronous to asynchronous teaching.

Many teachers do decide to use online technology for testing and extensive discussion board assignments, while keeping the in class environment rich with in-depth debates, experiential learning, concept critiques, team presentations, hands-on experiments, and demonstrations (Van Doorn and Van Doorn, 2013). Teaching f2f may be a preferred traditional delivery method for various teachers, but quick Internet access to information through web facilitated technology tools have enhanced versatility. Teachers can play classic experiments found on Youtube.com and provide website links to current event topics and embed them into their PowerPoint presentations presented on in class Wiimote interactive whiteboards like Smart Board (Sacks and Jones, 2011). Bring your own device (BYOD) policies encourage faculty, staff, and students to use their technology on campus and explore current events and utilize Cloud servers for data sharing and storage. On the other hand, technology-savvy students, and professors have found that convenience and economical factors (fewer trips to school) are some of the driving forces behind implementing more technology tools in web-facilitated courses.

Different teaching delivery formats have strengths and weaknesses and may well map to different learning needs of student cohorts (See Table 1). For example, it is far more difficult to have a genuine spontaneous sense of humor about a course topic in a purely asynchronous online format, but it is much more likely in f2f formats. Conversely, faculty preplanning, and organization of online courses is very intensive and complex, requiring several weeks of notice before being assigned to teach a completely online course for best design quality. Although, in comparison to f2f classroom structures, it is may be easier for a teacher to effect an extension change for an assignment by a few hours in an online course environment.

### Online course format

While the traditional f2f classroom setting provides many robust benefits, the fully online course format is quickly offering students and teachers flexibility with more course offerings and university applications for access to assignments on mobile platforms (e.g., cell phones, e-readers, iPads). Online portions of courses may be designed with dedicated pre-class PowerPoint lectures with voice over narration for enhanced auditory learning. The challenges to teachers may include staying up-to-date on course delivery software, grade book programs, and CMS training. Educators realize that learning is progressive and find that embracing new technology tools for their courses is a smoother transition when institutions provide continuous training and support them, as core faculty service providers, into the competitive, technological learning environments.

Online courses can meet student needs for economy and efficiency. Most experienced teachers realize that weekly quizzes remain one of the best motivational tools to prompt students to keep up with voluminous, but necessary readings. Research indicates that use of online quizzes may enhance scaffolding learning outside the classroom and encourage student reading motivation (Anthis and Adams, 2012). Full-time, non-traditional student workers with family obligations may find the quiet hours, late into the night, provide needed time to participate in discussion boards, prepare assignments, and answer questions with teachers via live interactive webinars (Van Doorn et al., 2012). Teachers and students alike have found that successful performance with online courses requires very intensive and lengthy sessions sitting at the computer terminal. If a laptop, iPod®, or iPad® is used with good Wi-Fi access, the classroom environment has no boundaries when the class is enriched with these interactive features. Even social science research is more efficient with use of Surveymonkey.com, Qualtrics.com, and SPSS statistical analysis software. Additional online technology tools include rubric assessment grading on Livetext.com and CMSs like Blackboard, readings on eReader devices, news media videos like NBC Learn and 60 min, National Geographic educational videos, instructional Youtube.com videos, and counseling session simulations with virtual Avatars from Second Life software (Sacks and Jones, 2011). Smartphones as mobile platforms have rapidly made inroads into the educational arena with creative applications that give students instant access to their courses (Smith, 2014). Research indicates that 73% of adults who are online use social media with Facebook as the top choice for communication (Duggan and Smith, 2013). There are, however, some limitations to student use of mobile platforms including security concerns around test administration.

Academic integrity can be compromised with online testing. While in class exams tend to lower cheating behaviors, universities have addressed some of these academic integrity problems for online courses with techniques like Remote Proctor which utilizes biometrics (fingerprinting and facial recognition), optical cameras and audio detection (no phone use allowed during exam), and the zone alarm tool (virtual box surrounding test taker which sounds an alarm if another person enters the testing space), and TurnItIn.com. Techniques like these may help to give instructors the confidence that online test taking is as secure, maybe even more so, than traditional in-class proctoring. Indeed, there may be pedagogical reasons for allowing an online, remotely proctored exam where students can type and reflect on answers that may prove superior against cheating as compared to traditional take-home exams. Overall, web-facilitated and blended/hybrid courses may have strengths beyond the traditional f2f delivery format.

Subsequently, administrators may want to consider collaborative, participative leadership styles that incorporate more faculty-driven pedagogy into training workshops and design selection of CMS. For example, the Quality Matters Program (QM) offers professional faculty-centered workshops and training based on designing fully online and blended courses to a master rubric (Quality Matters Program, 2011). These workshop resources, strategies, and tips from practiced online teachers may help new online teachers better navigate design delivery and lower their preparation time (Quality Matters Program, 2011; Neff, 2013). A continuum exists between how technology savvy workers are from the labels of a “Luddite” to a “Geek,” *viz.*, less to higher technologically skilled (Dunn et al., 2011a,b). Technology can be expensive and is not affordable to all seekers of higher education. U.S. News and World Report (“Best online,” 2014) rated the best online bachelor's programs based on methodologies of faculty credentials and training, student services and technology, and student engagement. Many online degree programs are taught in 8- to 12-week terms vs. the traditional 16-week semester. These trends need further research to assess course term lengths and respective student mastery of complex subjects, whether there is enough learning time for knowledge transfer efficacy. Educational institutions that provide access to updated computer labs for teachers and students to train and to do classroom assignments, respectively, may help to bridge disparities in online design, delivery, and quality performance across fully online and blended delivery formats.

In summary, when the academic goal of knowledge transfer and learning is foremost, the hybrid/blended format, with its online segment, is a great addition to the pedagogical teaching tools arsenal. The use of the online environment preserves valuable classroom time for those activities which are best accomplished in class, while providing a platform and set of tools that are in some ways superior to that of the traditional classroom setting. Our distinction between traditional and non-traditional student learning styles found in Table 1 is a useful one in order to understand the appropriate and productive use of these pedagogical tools and structures for higher learning. For the traditional undergraduate student who may be introverted or reticent to participate openly in a large class section like Introduction to Psychology, the online discussion board would allow a more accessible setting, and encourage such participation that otherwise might not occur. Available online support services and tools are at times critical for the non-traditional student living far from campus and pressed for time in retrieving reserve reading materials from a limited-hours campus library. On the other hand, community libraries are offering access to expensive computer and video equipment technology like 3-D printers, iPads, and music video equipment in studios that helps to offset the access and affordability inequities in training new skills (Humphrey, 2014).

## Discussion

From this literature review, we hope we created a useful traditional and non-traditional student typology of learner needs for referencing in higher education. Also, we presented several pedagogical recommendations for educators to consider for enhancement of knowledge transfer efficacy in addition to our traditional and non-traditional student needs typology table that includes improving curriculum assessment rigor and consideration for faculty-matched delivery formats. Specifically, by addressing curriculum rigor through the mapping of American Psychological Association (2007, 2013) guidelines and principles to SLOs, matching delivery formats and support services to traditional and emerging, non-traditional student learning needs, and improving technology training for core service providers to reach higher quality online instruction and student performance may together result in higher knowledge transfer efficacy. Also, our review of traditional f2f, web-facilitated and blended/hybrid, and fully online course delivery formats with respective teaching techniques may help to distinguish nuanced teaching and learning differences in these delivery modes. With higher dropout rates among non-traditional students, programs like CUNY's Accelerated Student in Associate Programs (ASAP) are showing remarkable improvements from a low 23% to higher 56% graduation rate on the first two cohorts followed. CUNY's program addresses retention concerns through improving support systems that address advising, student money issues, textbook costs, and flexible morning, afternoon, or evening class schedules (Kirp, 2014). Foremost, ASAP supports many of our typologies of the non-traditional student (Table 1) and their learning needs (Clark et al., 2002; Dobransky and Frymier, 2004; Myers, 2004).

Research suggests that web facilitated and blended/hybrid course formats may have the greatest advantages than either purely in-class or online formats for the non-traditional student cohort. The non-traditional cohort is more focused on learning mastery, pressed for time, and appreciative of a course that is “flexible and well-organized” (Hoyert and O'Dell, 2009). Properly designed and executed by trained teachers, the blended/hybrid format may best fulfill these needs. With research suggesting the blended/hybrid course format is high on learning efficacy, educational institutions may need more funding support to assist faculty training on online asynchronous and synchronized platforms like *Wimba* and *Collaborate*, and for provided classroom space for group learning to maximize concept and skill demonstrations. An implication from this review suggests marked differences and retention results are occurring between f2f, blended/hybrid, and fully online courses and may be associated with the respective traditional and non-traditional learning typologies. Therefore, we hope the learning styles typology Table 1 may serve as a guide and working reference tool for teachers and administrators who are planning and designing quality courses. Also, consideration of mapping American Psychological Association (2007, 2013) guidelines and principles to SLOs may help guide curriculum committees on designing rigorous assignments for improving student learning. Additional value-added features may include better standards for social science program accountability and improved student performance data for accreditation purposes.

Implications from our new typology model of traditional and non-traditional student needs are expressed in terms of suggestions for future changes in university support, resource allocations, and faculty and student training. The four cohorts of traditional undergraduates and graduates and non-traditional undergraduates and graduates have specific implications presented in Table 1. First of all, traditional undergraduates may continue to need daytime classes that are spread out during the week (to accommodate many on-campus and extra-curricular activities), but in the future, they will likely require enhanced support for in class BYOD strategies, supplemented by on-line, blended work, to save valuable classroom time for active teaching and learning. Going forward, instructors of these students should be trained in, and supported with a variety of classroom technology that seamlessly integrates video, sound and live lectures in a captivating manner. Traditional undergraduates will also want and expect interaction with faculty outside of class as faculty club sponsors and mentors. Therefore, the university may need to adjust expectations of teaching loads. The large university two-tier system, where core faculty work primarily as researchers and other faculty and graduate assistants are student-oriented, will likely persist. Secondly, non-traditional undergraduates will need greater support for both training in classroom technology and workshops for relearning cognitive learning and study skills upon their return to the academy. We reemphasize that universities should set-aside computer, writing, and math labs with tutors that are open up to 24 h per day. The non-traditional undergraduates will need more flexibility with night and weekend course offerings built around their work and family obligations. Faculty may need to regularly coach and retrain traditional and non-traditional students, respectively, on robust cognitive study skills (mnemonics, visual imagery, and word associations), goal-setting theories (Locke and Latham, 2002), and self-efficacy (Bandura and Locke, 2003) for enhancing knowledge transfer and achievement goals during their academic journey, and possibly higher retention rates.

While undergraduate students have particular needs, the traditional graduate students have needs for more opportunities to work closely with specific faculty mentors in research projects, academic apprenticeships and teacher training. This cohort needs access to state-of-the-art research laboratories, databases, and libraries. Traditional graduate students should have the inclination to pursue international and collaborative research projects; therefore, the university should seek more foreign-university and research institute cooperation agreements. Because of prior training, recent graduation from college, and desire for more research and writing time, on-line courses and maybe the blended/hybrid and/or flipped classroom, would be most applicable to this group, but clearly, more research is needed here. Finally, the non-traditional graduate cohort in mid-career have their unique student needs and expectations. Non-traditional graduate students tend to require more hands-on, goal-directed and pragmatic, career-oriented learning. In addition to standard lectures, well-designed experiential/kinesthetic and collaborative learning strategies such as role-play, cultural story-telling styles, and academic simulations will benefit students who learn best by doing. From our research, we suggest that this cohort will benefit from fewer on-line courses than traditional graduates, and possibly seek more blended and in-class course combinations. Professors who design their courses with a great deal of front-end organization, whose expectations are very clear, yet flexible in terms of scheduling, may be able to better accommodate non-traditional students life event occurrences, such as military deployments, family roles, and possible elder care responsibilities. Similar to the instructors of non-traditional undergraduates, the teachers of non-traditional graduates will need to be well-trained and experienced in transferring high levels of knowledge in an efficacious manner, again using a combination of streamlined lecture and experiential learning techniques in a live classroom setting. Clearly, both the learning needs of these four distinct student cohorts and the abilities and training of the faculty/instructors who will be charged with knowledge transfer responsibility to meet their learning needs, are very distinct. It is our hope that we have presented a timely typology, and important research agenda for future scholarly work.

In addition, we call for the academy to be cautious of a one-size-fits-all approach, and more closely align resources and expectations with the needs of these different student cohorts. Our category G. *Course Subject Needs* is a preliminary list that needs further research to indicate which discipline courses may benefit from specific delivery modalities. With this typology educational institutions may be able to formulate better strategies to serve traditional and non-traditional student cohorts and provide enhanced rigor to their programs. Although our typologies on learning needs are significantly supported by research findings, it does have limitations. First of all, comparatively, it doesn't address as many of the graduate student learning styles. The categories consisting of needs for institutional support, computer technology, and educational culture and social activities call for further investigation, especially as the educational experience evolves. Therefore, future research could address the comparisons between undergraduate and graduate student learning styles and the instructor course preparation techniques that maximize efficient and productive learning.

Moreover, our research review revealed an immediate urgency felt by educational administrators to address lower reported retention indicators. Using our learning needs typology as a tool to match quality teaching to the traditional and non-traditional student learners may help to reduce drop out rates and increase retention. We consider this early evidence of a “bounce back” phenomenon that may be occurring when students attempt online learning, suffer poor performance, and then drop out (or hopefully, they decide to proceed back into the f2f classrooms to finish their program requirements toward graduation). Overall, this phenomenon suggests that the traditional in class format of brick and mortar schools may still set space and place for higher performance and learning, when viewed over a student's entire educational program. More behavioral research is needed to address pedagogical delivery methods, these online student experiences, behaviors, and institutional support experiences. Recent research on advisement duty by faculty and academic support staff was found to be an important part of the educational social process, where monitoring and mentoring may improve student institutional commitment (IC) (Beck and Milligan, 2014). Further support found advising as a procedural process that monitors and evaluates students' satisfactory performance, knowledge mastery, and guides the student to completion of degree program courses. As a result, students find they meet their successful, graduation goals (Kirp, 2014).

Further implications from our research review include institutional acknowledgement of present and future teacher and student training needs, especially with continuous support services during rapid advances in educational technology. Administrators must consider best fit options for teachers based on their knowledge, skills, and abilities and their training for matching them to their preferences and most productive course delivery methods, whether in f2f classes, 100% online courses, and/or blended/hybrid courses. Moreover, social networks are where Americans spend 23% of their online time with Internet service (Nielsen Social Media Report: Q3, 2011). Therefore, educational administrators may need to consider how best to facilitate some faculty-led strategies for pedagogical training of “best practices” for course delivery formats, stronger social science curriculums, the use of social media, and adult learner continuing education (Caffarella and Daffron, 2013). Opportunities exist for teachers to easily incorporate language assignments (Mango Languages, 2011) and even study abroad opportunities to help build multi-lingual abilities and globally confident, ready students with worldview perspectives. Setting higher student performance goals and training innovative online teaching methods may help to broaden student job skills, enhance diversity knowledge through inclusive teaching, and promote lifelong career marketability for students. The efficacious transfer of knowledge to 21st century students is important for facilitating higher performance, and overall, student mastery of disciplinary content and should be a part of the Academy's focus.

## Author biographies

Dr. Judy R. Van Doorn, is an Assistant Professor of Psychology—Industrial Organizational psychology. She teaches in the Psychology department at Troy University, Phenix City, Alabama 36869, USA. Her research interests include teaching techniques in psychology, workplace psychology, environmental psychology, conservation behaviors, leadership, self-concept, values, aesthetics, and creativity. Dr. John D. Van Doorn is an Assistant Professor of Political Science—International Relations. He teaches in the Political Science department at Troy University, Columbus/Ft. Benning, Georgia 31905, USA. His research interests include online teaching techniques, comparative politics, international foreign policy, international environmental law, leadership, and comparative democratization.

### Conflict of interest statement

The authors declare that the research was conducted in the absence of any commercial or financial relationships that could be construed as a potential conflict of interest.
